# Insulin Adherence in Type 2 Diabetes in Mexico: Behaviors and Barriers

**DOI:** 10.1155/2018/3190849

**Published:** 2018-07-15

**Authors:** Janneth Bermeo-Cabrera, Paloma Almeda-Valdes, Josefa Riofrios-Palacios, Carlos A. Aguilar-Salinas, Roopa Mehta

**Affiliations:** ^1^Departamento de Endocrinología y Metabolismo, Instituto Nacional de Ciencias Médicas y Nutrición Salvador Zubirán, Mexico City, Mexico; ^2^Unidad de Investigación de Enfermedades Metabólicas, Instituto Nacional de Ciencias Médicas y Nutrición Salvador Zubirán, Mexico City, Mexico

## Abstract

**Objective:**

To investigate factors associated with insulin adherence in subjects with type 2 diabetes mellitus (T2D) attending a tertiary care centre in Mexico City.

**Material and Methods:**

Cross-sectional study, including 200 patients. Adherence to insulin therapy was measured with a medication adherence questionnaire. Sociodemographic data and factors related to insulin omission were collected and compared between the nonadherent and adherent groups.

**Results:**

We categorized 117 (58.5%) patients as nonadherent and 83 (41.5%) as adherent. Among the adherent, only 22 patients (11%) had excellent adherence to insulin therapy. The following factors were associated with nonadherence: lack of planning of daily activities (46.1%), fear of hypoglycemia (41%), economic factors (15.4%), and number of insulin applications (2.31 versus 1.76 applications per day).

**Conclusions:**

In this study, patients with type 2 diabetes attending a tertiary care referral centre showed inadequate adherence to insulin therapy. The principal factors associated with insulin omission were low socioeconomic status, fear of hypoglycemia, and a greater number of insulin applications per day.

## 1. Introduction

In their treatment guidelines, both the American Diabetes Association and the American Association of Clinical Endocrinologists confirm that insulin (either for initiation in patients with severe hyperglycemia or for treatment intensification) is the most efficient glucose-lowering agent [1, 2]. Despite this, insulin use remains relatively low. In the United States, the National Health and Nutrition Examination Survey (2005–2012) reported that the proportion of patients with diabetes on any insulin (includes insulin only and insulin plus oral diabetes medications) was 29.1% (95% CI 26.7–31.5) [3]. Rebolledo and Arellano comment that the usage rate of oral diabetes medications among adults with type 2 diabetes has remained almost three times higher than that of insulin (50.3 versus 17.8%) [4]. In Mexico, the most recent National Health Survey showed that even though insulin use has increased, it is still inadequate; between 2012 and 2016, the prevalence of insulin plus oral medication increased from 6.6% to 8.8%, while that of insulin only regimens increased from 6.5% to 11.1% [5]. Among the barriers associated with insulin use (including patients' perceptions regarding insulin safety, cultural beliefs, social factors, health literacy, medication costs, and physician-related attitudes), patient noncompliance is commonly encountered [6]. Insulin nonadherence may be considered in terms of primary nonadherence (failure to fill pharmacy prescriptions) or poor persistence due to insulin dose omissions, either deliberate or accidental [7]. Peyrot et al. have explored factors related to insulin omission/nonadherence. In the Global Attitudes of Patients and Physicians in Insulin Therapy for Diabetes Mellitus (GAPP) Survey, 8.6% of subjects omitted insulin doses because of pain associated with the injection [8]. Almost half of the participants (43.3%) omitted injections because they felt that insulin interfered or was intrusive in their daily activities. The perception of insulin injections as painful or embarrassing was also associated with dose omission, as were a large number of daily injections. Unsurprisingly, in those reporting insulin omission, glycemic control was inadequate with elevated glycated hemoglobin (A1c) levels.

The diabetes attitudes, wishes, and needs (DAWN) study also evaluated patient attitudes towards insulin therapy [7]. Patients rated the efficacy of insulin as low, and about a quarter believed that insulin would not help them. Many blamed themselves for having to initiate insulin therapy and reported high degrees of diabetes distress.

In the Hispanic-Latino population, patients with diabetes often show reluctance to start insulin therapy, frequently due to myths and misconceptions about its use [9]. In a systematic review, Rodríguez-Gutiérrez et al. reported that the main concerns regarding insulin use included the following: that it is associated with blindness, indicated more advanced disease, a punishment, difficult to administer, expensive, and more time-consuming to manage [10]. Herrero and Caballero found that those patients with such beliefs had a poorer perception of their health, were more likely to have insulin omission, had greater emotional distress, and generally showed lower treatment adherence [11].

In Mexico, Lerman et al. reported that insulin therapy was delayed; the mean duration of type 2 diabetes (T2D) at insulin initiation was 14 years [9]. In addition, 41% of patients were nonadherent to insulin therapy. This was more common in women and was associated with depression and a negative attitude about insulin. Patients felt that insulin therapy was required because their disease had worsened due to their failure in treatment adherence. Patients also mentioned a fear of injections and felt that insulin would restrict their lifestyle. Others believed that insulin was associated with the development of diabetic complications. These attitudes were most common in lower income subjects and those with less diabetes-related knowledge. To our knowledge, there are no studies evaluating adherence to insulin therapy in Mexico.

The aim of this study is to evaluate the adherence to insulin therapy in patients with T2D treated at a large tertiary care centre and to identify the factors associated with omission or nonadherence to the insulin regimen.

## 2. Methods

We performed a cross-sectional study in patients with T2D attending the diabetes clinic of a tertiary care centre in Mexico City. In the diabetes clinic, there are approximately 2800 patients with type 2 diabetes, 878 of which have been prescribed insulin treatment. Between March and December 2017, 200 patients were recruited utilizing convenience sampling. All participants were adult subjects under treatment with at least one insulin dose per day over the last 3 months (i.e., a stable dose of insulin). Patients were recruited the day of their appointment and underwent a personal interview. Sample size was estimated considering an alpha error of 0.05 and a precision of 0.05 to detect a 15% prevalence of nonadherence. This study was approved by the Comité de Ética del Instituto Nacional de Ciencias Médicas y Nutrición Salvador Zubirán (study reference number 1967). Informed consent was obtained from all participants. Patients were excluded if they had type 1 diabetes mellitus, a secondary cause of diabetes, or had been admitted to hospital for an acute illness a month prior to commencing the study. In all patients, a brief medical history and current laboratory results were obtained. The questionnaires for evaluating adherence and related factors were self-administered with supervision from one of the study investigators. Participants completed the questionnaires in a quiet room and were given sufficient time to do so.

The main outcome was adherence or nonadherence to insulin therapy, evaluated using the 8 item Morisky-Green Questionnaire [12, 13]. A score of 8 indicated excellent adherence; 6 to <8, moderately good adherence; and <6 was indicative of poor adherence.

Factors associated with omission were sought using a questionnaire formulated using evidence from previous publications [7, 9] and a pilot study carried out at our centre. A double-interview method was used. Consecutive patients were recruited to complete the 23-item questionnaire (*n* = 10). Participants were asked to explain what each item meant and why they chose a particular response. The questionnaire was adapted taking into account the discrepancies, namely, what was intended and what was understood. In a second step, the revised questionnaire was tested in a further 10 patients whose results suggested good acceptability of this instrument.

The following three aspects associated with insulin adherence were evaluated: interference with daily activities, experience with insulin injections, and economic factors. The instrument contained 23 questions with answers captured on a 5-point Likert scale. The responses were analyzed in a dichotomized fashion (using chi-squared test). The extremes of the scale, that is, the percentage of patients answering totally agree + agree, were considered as one group, while, completely unsatisfied + unsatisfied all of the time + a lot of the time constituted the comparison group.

In the statistical analysis, patients who had excellent adherence or moderately good adherence to insulin therapy (≥6 in the Morisky-Green Questionnaire) were compared to those who had poor adherence (<6 in the Morisky-Green Questionnaire). The distribution of continuous variables was assessed by the Kolmogorov-Smirnov test. Continuous variables were compared between adherence groups using the Student *t*-test (parametric) or the Mann–Whitney *U* test (nonparametric). The chi-squared test was used to analyze categorical variables. A multivariate regression model was generated with adherence as the dependent variable (Morisky score) and the factors identified in bivariate analysis as independent variables. A *p* value of <0.05 was considered as statistically significant. All analyses were performed using SPSS version 21.

## 3. Results

Of the T2D patients under insulin treatment, 200 consecutive subjects fulfilled the selection criteria and were included in this study. The majority of participants were women (65%), the mean age was 61.5 ± 12.0 years, the mean duration of diabetes was 19.6 ± 8.7 years, and the median time since insulin initiation was 6 (3.25–10) years. Excellent adherence (Morisky score of 8) was found in only 11% (*n* = 22), moderate adherence in 30.5% (*n* = 61), and low adherence in 58.5% (*n* = 117).

Table 1 shows the characteristics of the population according to 2 adherence categories (adherent versus nonadherent). The “adherent” group was formed by combining those subjects with excellent adherence with those showing moderate adherence (*n* = 83 subjects); this was compared with the “nonadherent” group (Morisky score ≤ 5) (*n* = 117). There was no gender difference between these groups (*p* = 0.359). The age of participants in the adherent group was significantly greater than the age those in the adherent group (64 versus 59 years, respectively, *p* = 0.037). The age at diagnosis of diabetes was similar between groups (41.8 versus 42.1 years, *p* = 0.502). Regarding the duration of diabetes, this was significantly longer in the adherent group (20 versus 17.5 years, *p* = 0.022). With respect to the median time since insulin initiation, no differences were found between the groups (5 versus 6 years, *p* = 0.685). There was no difference in the education level between groups (*p* = 0.536), approximately 30% of participants had less than middle school education.

The characteristics of both groups with respect to insulin treatment are shown in Table 2. A simple insulin regimen (only basal insulin) was present in 62.5% of the population. There was no difference between groups with respect to the use of only basal insulin (NPH and basal analogues, *p* = 0.136). The commonest basal insulin was NPH (NPH 65.3%, glargine U100 28.6%, and degludec U100 6.1%). However, when comparing adherence groups, basal insulin analogues were more frequently used in the adherent group (42.7 versus 28.9%, adherent versus nonadherent group, resp., *p* = 0.046). Insulin intensification regimens were present in the remaining subjects. The use of premixed insulin was rare (2%). No subject utilized GLP-1 agonists. Basal-bolus and basal-plus regimens were predominant, recorded in 35.5%. Regular insulin was the most prevalent preprandial agent (90.1%). In the nonadherent group, a significantly higher number of patients were on such complex regimens (25.3 versus 42.7%, adherent versus nonadherent groups, resp., *p* = 0.011). Insulin dose was significantly higher in the nonadherent subjects (0.40 [0.28–0.54] versus 0.54 [0.37–0.73], *p* < 0.0001). As one would anticipate, the number of insulin applications was also higher in the nonadherent group (1.76 ± 0.79 versus 2.31 ± 0.96, *p* < 0.0001). The use of prefilled insulin pen devices was significantly higher in the adherent group (31.3 versus 17.9%, *p* = 0.028).

The biochemical characteristics according to adherence category are shown in Table 3. The nonadherent group had a significantly higher A1c compared to the adherent group (8.4 versus 8.9%, *p* = 0.024). The levels of fasting plasma glucose, triglycerides, and LDL cholesterol did not show differences between groups.

### 3.1. Interference with Daily Activities

The adherent group reported that they planned their daily activities around the application of insulin (58.7% versus 53.9%, adherent versus nonadherent, resp., *p* = 0.012) (Table 4). This group also reported greater interference with meals (*p* = 0.001) and exercise (*p* = 0.003). There was no difference between groups regarding interference with social and recreational activities, sexual activity or work, and professional development.

### 3.2. Experience with Insulin Injections

The second factor evaluated was subject experience with regard to the application of insulin (Table 5). Persons who were nonadherent felt that injecting was the most difficult part of their treatment (19.0% versus 21.4%, adherent versus nonadherent, resp., *p* = 0.044). This group reported pain and bruising more frequently than the adherent group (33.7% versus 59.8%, *p* = 0.002 and 32.5% versus 55.5%, *p* = 0.002). The nonadherent group also felt greater embarrassment with insulin application (8.4% versus 25.7%, *p* = 0.033). Finally, the most significant difference between adherence groups was fear of hypoglycemia; here again, the nonadherent subjects more frequently reported fear (28.9% versus 41.0%, *p* < 0.0001).

### 3.3. Economic Aspects

The final aspect examined was economic reasons for insulin omission (Table 6). The nonadherent group reported that this was an important factor all the time or almost all the time (9.6% versus 15.4%, adherent versus nonadherent, resp., *p* < 0.0001). Health insurance or access to social security was significantly less prevalent in the nonadherent group (51.8% versus 35.9%, *p* = 0.025). Economic support from relatives was also more commonly cited in the nonadherent subjects (26.5% versus 37.6%, *p* = 0.012). Finally, inadequate beliefs surrounding insulin application were equally present in both adherence categories (*p* = 0.116).

A linear regression model was constructed to determine factors independently associated with adherence. The dependent variable was the adherence score. The independent variables were age, use of needle and syringe, planning of activities around the application of insulin, fear of hypoglycemia, and economic factors. The factors significantly associated with better adherence were adequate economic resources, planning of daily activities around insulin application, lack of fear of hypoglycemia, and fewer insulin applications per day. This model is shown in Table 7.

## 4. Discussion

In this study, we describe the characteristics of insulin-treated patients in a tertiary care centre in Mexico City. We evaluated the level of insulin adherence and explored the factors related to nonadherence. About a third (30.5%) of subjects showed moderate adherence to insulin therapy and excellent adherence was infrequent (11%).

In our population, one important characteristic was that the average duration of diabetes was almost twenty years; however, the mean number of years on insulin therapy was only six, suggesting a significant delay for commencing insulin. Lerman et al. have shown similar findings in a Mexican population; at the time of insulin initiation, the mean duration of type 2 diabetes was 14 ± 9 years and the mean A1c concentration was 10.8 ± 1.4% [9]. Rodríguez-Gutiérrez et al. reported that inappropriate beliefs (negative attitudes towards insulin therapy, psychosocial barriers, and myths regarding insulin use) are an important barrier for physicians when trying to initiate and sustain insulin treatment (persistence of treatment) in this region [10]. Available information regarding insulin adherence in Latin American countries is scarce, and Lerman et al. reported that 41% of Mexican patients did not adhere to insulin treatment [9]. In our population, 58.5% were nonadherent (when this was considered as a dichotomous variable).

Regarding factors associated with nonadherence, both age (64 versus 59 years, adherent versus non adherent, resp., *p* = 0.037) and mean duration of diabetes (20 versus 17 years, *p* = 0.02) were significantly lower in the nonadherent group compared to the adherent group. There is no clear explanation for this finding. We can speculate that older patients and patients with a longer disease duration may have better adherence because they have learnt to accept the disease to a greater extent than younger patients or patients with recent diagnosis. In the Global Attitudes of Patients and Physicians in Insulin Therapy (GAPP) study, 34.6% of the study population admitted to omitting their insulin dose at least once in the previous month. Insulin omission was more frequent among women (50.8%), and age and duration of diabetes were lower in the low adherence group (50 and 6 years, resp.) [8]. Peyrot et al. found similar results in an internet survey in 502 diabetic individuals, 388 of which had T2D [14]. Intentional insulin omission was identified in 20% of participants. Sociodemographic factors associated with insulin omission were younger age, lower income, and higher education level. In our study, we did not find differences in adherence associated with educational level (*p* = 0.536).

In the nonadherent group, the number of insulin applications per day was significantly higher (*p* < 0.0001). In addition, a higher proportion of nonadherent individuals applied insulin using needle and syringe compared to the adherent group (68.7% versus 82.1%, adherent versus nonadherent, resp., *p* = 0.028). Slabaugh et al. in a retrospective analysis of Medicare data reported that adherence-adjusted probability (proportion of covered days ≥ 80%) was positively associated with pen device use (OR 2.19; 95% CI 1.86–2.59) [15]. The nonpersistence-adjusted risk was significantly lower (58%) in the cohort using pen devices compared to those using syringes (HR 0.42; 95% CI 0.38–0.45). The simplicity of use and lower pain associated with pen devices probably promotes improved compliance [16].

In our study, the nonadherent subjects did not plan their activities around insulin application. They also believed that insulin did not interfere with meals or exercise. In the GAPP study subjects who omitted insulin reported interference with daily activities (43.3%), this difference suggests that subjects who omit insulin in our centre do not consider insulin treatment as an integral part of their daily lives [8].

With respect to the experience associated with insulin injection, the nonadherent group reported that fear of hypoglycemia, pain, bruising, embarrassment, and injecting insulin were considered the most difficult parts of treatment. Ross et al. identified anxiety related to disease progression, fear of hypoglycemia, embarrassment, panic of injections, regimen complexity, and fear of weight gain as the factors most strongly associated with insulin omission in Canada [17]. In the GAPP study, 28.9% omitted insulin because of pain related to injections [8]. Peyrot et al. reported that the most important factors were a greater number of injections per day, interference with daily activities, pain, and embarrassment related to insulin injection [14]. Farsaei et al. reported the following factors associated with insulin noncompliance: time-consuming application of insulin, injection site pain, embarrassment, forgetfulness, feeling worse after insulin injection, difficulties in injecting, concurrent illness, insulin shortage, cost, and weight gain associated with insulin [18].

Finally, with respect to economic factors, a lower proportion of nonadherent subjects had health coverage or economic support from their families. In Mexico, diabetes treatment is often an out of pocket expense and insulin treatment is perceived as costly [19]. Farsaei et al. reported that medication cost had a significant relationship with low compliance in a similar population [18].

Although there is extensive worldwide literature regarding patient barriers to insulin therapy and nonadherence, there is little information on interventions to improve adherence to insulin [20]. Possible actions to address the principle factors include the use of newer therapies, in particular insulin analogues, and pen devices instead of needle and syringe for insulin administration. Insulin analogues are associated with a lower risk of hypoglycemia, in particular nocturnal hypoglycemia [21, 22]. In a systematic review investigating real-world factors affecting adherence to insulin, Davies et al. found that switching or initiating insulin administered by a pen device improved adherence in four of five studies investigating this factor. They also suggested that a more flexible insulin regimen (e.g., use of newer insulin analogues) would impact the perceptions of interference with daily activities and may result in fewer applications per day. Finally, these authors comment that adherence is improved when the financial burden to the patient is reduced [23]. Education regarding the prevention and treatment of hypoglycemia is also necessary to allay patient fears. Lerman et al. showed that a support from a diabetic nurse specialist was a positive predictor for adherence [9]. Improving access to health care services is also necessary.

The results of this study may help to establish actions to confront and improve the lack of adherence to insulin treatment in patients with T2D. Health care providers should be encouraged to address the factors identified in this study in the consultation. Doggrell and Chan found that the best strategy was to involve patients in all aspects of the decision-making process around insulin therapy [20]. Finally, the implementation of education programs, patient empowerment, and specific interventions targeting young and recently diagnosed individuals (possibly utilizing newer technologies or social media) may be worth considering for improving insulin adherence [19].

Some limitations of the present study should be acknowledged. Only the most important factors reported in other studies were evaluated; this was not a totally inclusive search. Insulin omission was evaluated through questionnaires and not directly. Patient self-reporting has previously been shown to either underestimate or overestimate adherence [24]. The impact of participating in a study with researchers present at the time of questionnaire completion may have influenced the way participants responded. To minimize this limitation, questionnaires were completed anonymously and patients were encouraged to be honest knowing that the answers would not affect subsequent patient care. Factors related to health care providers were not evaluated, and the study was performed in a referral centre; thus, results might not be generalizable. Finally, insulin treatment is only one component of the integral treatment in patients with T2D. This study did not evaluate other components of the treatment (e.g., number of concomitant medications).

## 5. Conclusions

In this study, excellent adherence to insulin was identified in only a minority of patients with T2D. The principal factors associated with insulin nonadherence were fear of hypoglycemia, interference with daily activities, a greater number of applications per day, and economic factors. In order to address these findings, education programs designed to tackle these issues in a multidisciplinary team setting should be considered.

## Figures and Tables

**Table 1:** Characteristics of study participants, classified by adherence category.

Variable	Nonadherent *n* = 117	Adherent *n* = 83	*p*
Gender			0.359
Women	73 (62.4)	57 (68.7)	
Men	44 (37.6)	26 (31.3)	
Age, years	59 [53–69]	64 [54–73]	0.037
Weight, kg	71.7 ± 14.9	70.5 ± 10.9	0.551
Age at diagnosis of diabetes, years	42.1 ± 11.2	41.8 ± 11.6	0.502
Duration of diabetes, years	17.5 [12–25]	20 [15–26]	0.022
Time since insulin initiation, months	72 [42–120]	60 [36–120]	0.685
Education level			0.536
Incomplete primary	19 (16.2)	9 (10.8)	
Primary/elementary	17 (14.5)	13 (15.7)	
Secondary/middle school	39 (33.3)	32 (38.6)	
High school	13 (11.1)	12 (14.5)	
College	24 (20.5)	11 (13.3)	
Postgraduate	5 (4.3)	6 (7.2)	

Data are expressed as means ± standard deviation, median [interquartile range], or number (%).

**Table 2:** Insulin regimen characteristics according to adherence category.

Variable	Nonadherent *n* = 117	Adherent *n* = 83	*p*
Insulin applications/day, number	2.31 ± 0.96	1.76 ± 0.79	<0.0001
Insulin dose, units/kg	0.54 [0.37–0.73]	0.40 [0.28–0.54]	<0.0001
Form of insulin application			0.028
Pen	21 (17.9)	26 (31.3)	
Syringe	96 (82.1)	57 (68.7)	
Basal insulin			0.046
NPH	81 (71.1)	47 (57.3)	
Basal insulin analogue (glargine U100 and degludec U100)	33 (28.9)	35 (42.7)	
Preprandial insulin			0.611
Regular	44 (88.0)	20 (95.2)	
Rapid acting insulin analogue (lispro and aspart)	6 (12.0)	1 (4.8)	
Basal-bolus or basal-plus regimen	50 (42.7)	21 (25.3)	0.011

Data are expressed as means ± standard deviation or number (%).

**Table 3:** Biochemical parameters of the participants according to adherence category.

Variable	Nonadherent *n* = 117	Adherent *n* = 83	*p*
Glucose, mg/dl	134 [96–181.5]	123.5 [88–170]	0.303
A1c, %	8.9 [7.9–10.4]	8.4 [7.5–9.6]	0.024
Triglycerides, mg/dl	136.5 [100–198]	131 [100–185]	0.771
Total cholesterol, mg/dl	171 [145–193]	166 [144–196]	0.711
HDL cholesterol, mg/dl	46 [38–55]	46 [41–54.5]	0.727
LDL cholesterol, mg/dl	98 [79.5–114.5]	98 [79–114]	0.938

Data are expressed as means ± standard deviation or median [interquartile range].

**Table 4:** Interference with daily activities according to adherence category.

	Nonadherent *n* = 117	Adherent *n* = 83	*p*
Do you plan your activities around insulin application?	63 (53.9)	57 (58.7)	0.012
Applying insulin interferes with your meals?	39 (33.3)	41 (49.4)	0.001
Applying insulin interferes with doing exercise?	34 (29)	37 (44.6)	0.003
Applying insulin has a negative impact in your social and recreational activities?	19 (16.3)	9 (10.8)	0.336
Applying insulin has a negative impact in your sexual activity?	15 (12.8)	6 (7.2)	0.487
Applying insulin has a negative impact in your work and professional development?	20 (17.1)	6 (7.2)	0.225

Data are expressed as number (%).

**Table 5:** Experience with insulin application according to adherence groups.

	Nonadherent *n* = 117	Adherent *n* = 83	*p*
Injecting is the most difficult part of the treatment	25 (21.4)	38 (19)	0.044
Injecting insulin causes pain	70 (59.8)	28 (33.7)	0.002
Injecting insulin causes bruising	65 (55.5)	27 (32.5)	0.002
Injecting insulin is embarrassing	30 (25.7)	7 (8.4)	0.033
Worry of having a hypoglycemia episode	48 (41)	24 (28.9)	<0.0001

**Table 6:** Economic factors and inadequate beliefs associated with the omission of insulin according to adherence groups.

	Nonadherent *n* = 117	Adherent *n* = 83	*p*
Do you omit your insulin for economic reasons?	18 (15.4)	8 (9.6)	<0.0001
Do you have social security or private health insurance?	42 (35.9)	43 (51.8)	0.025
Do you receive economic support from family to buy insulin?	44 (37.6)	22 (26.5)	0.012
Do you believe insulin causes health problems (blindness, amputation, kidney damage)?	13 (11.1)	7 (8.4)	0.116

**Table 7:** Linear regression model showing the factors associated with adherence (as quantified using the Morisky adherence score).

Variables	Coefficient beta (95% CI)	*p*
Planning of daily activities around the application of insulin	0.218 (0.122 to 0.449)	0.001
Number of insulin applications per day	−0.151 (−0.012 to −0.028)	0.023
Lack of fear of hypoglycemia	−0.128 (−0.492 to −0.139)	0.001
Lack of economic resources	−0.249 (−0.598 to −0.198)	<0.0001

Dependent variable: Morisky score. Included variables: age, use of syringe and needle, *R*^2^ = 0.234, *p* < 0.0001.

## Data Availability

The relevant laboratory/clinical data used to support the findings of this study are included within the article.

## References

[1] American Diabetes Association (2018). 8. Pharmacologic approaches to glycemic treatment: *Standards of Medical Care in Diabetes—2018*. *Diabetes Care*.

[2] Garber A. J., Abrahamson M. J., Barzilay J. I. (2018). Consensus statement by the American Association of Clinical Endocrinologists and American College of Endocrinology on the comprehensive type 2 diabetes management algorithm – *2018 executive summary*. *Endocrine Practice*.

[3] Selvin E., Parrinello C. M., Daya N., Bergenstal R. M. (2016). Trends in insulin use and diabetes control in the U.S.: 1988–1994 and 1999–2012. *Diabetes Care*.

[4] Rebolledo J. A., Arellano R. (2016). Cultural differences and considerations when initiating insulin. *Diabetes Spectrum*.

[5] Instituto Nacional de Salud Pública Encuesta Nacional de Salud y Nutrición de Medio Camino 2016. http://promocion.salud.gob.mx.

[6] Khunti K., Wolden M. L., Thorsted B. L., Andersen M., Davies M. J. (2013). Clinical inertia in people with type 2 diabetes: a retrospective cohort study of more than 80,000 people. *Diabetes Care*.

[7] Peyrot M., Rubin R. R., Lauritzen T. (2005). Resistance to insulin therapy among patients and providers: results of the cross-national diabetes attitudes, wishes, and needs (DAWN) study. *Diabetes Care*.

[8] Peyrot M., Barnett A. H., Meneghini L. F., Schumm-Draeger P. M. (2012). Factors associated with injection omission/non-adherence in the Global Attitudes of Patients and Physicians in Insulin Therapy study. *Diabetes, Obesity and Metabolism*.

[9] Lerman I., Díaz J., Ibarguengoitia M. (2009). Nonadherence to insulin therapy in low-income, type 2 diabetic patients. *Endocrine Practice*.

[10] Rodríguez-Gutiérrez R., Millan-Ferro A., Caballero E. A. (2015). Myths and misconceptions about insulin therapy among Latinos/ Hispanics with diabetes: a fresh look at an old problem. *Journal of Diabetes & Metabolism*.

[11] Herrero M. M., Caballero A. E. (2006). The presence of the blindness myth in Latino patients with type 2 diabetes. *Presented in the International Diabetes Federation Meeting*.

[12] Morisky D. E., Ang A., Krousel-Wood M., Ward H. J. (2008). Predictive validity of a medication adherence measure in an outpatient setting. *The Journal of Clinical Hypertension*.

[13] Osborn C. Y., Gonzalez J. S. (2016). Measuring insulin adherence among adults with type 2 diabetes. *Journal of Behavioral Medicine*.

[14] Peyrot M., Rubin R. R., Kruger D. F., Travis L. B. (2010). Correlates of insulin injection omission. *Diabetes Care*.

[15] Slabaugh S. L., Bouchard J. R., Li Y., Baltz J. C., Meah Y. A., Moretz D. C. (2015). Characteristics relating to adherence and persistence to basal insulin regimens among elderly insulin-naïve patients with type 2 diabetes: pre-filled pens versus vials/syringes. *Advances in Therapy*.

[16] Slabaugh S. L., Curtis B. H., Clore G., Fu H., Schuster D. P. (2015). Factors associated with increased healthcare costs in Medicare advantage patients with type 2 diabetes enrolled in a large representative health insurance plan in the US. *Journal of Medical Economics*.

[17] Ross S. A., Tildesley H. D., Ashkenas J. (2011). Barriers to effective insulin treatment: the persistence of poor glycemic control in type 2 diabetes. *Current Medical Research and Opinion*.

[18] Farsaei S., Radfar M., Heydari Z., Abbasi F., Qorbani M. (2014). Insulin adherence in patients with diabetes: risk factors for injection omission. *Primary Care Diabetes*.

[19] Lerman I. (2009). Barreras que dificultan la aplicación temprana de insulina en el paciente con diabetes tipo 2. *Asociación Latinoamericana de Diabetes*.

[20] Doggrell S. A., Chan V. (2015). Adherence to insulin treatment in diabetes: can it be improved?. *Journal of Diabetes*.

[21] Cahn A., Miccoli R., Dardano A., Del Prato S. (2015). New forms of insulin and insulin therapies for the treatment of type 2 diabetes. *The Lancet Diabetes & Endocrinology*.

[22] Horvath K., Jeitler K., Berghold A. (2007). Long-acting insulin analogues versus NPH insulin (human isophane insulin) for type 2 diabetes mellitus. *Cochrane Database of Systematic Reviews*.

[23] Davies M. J., Gagliardino J. J., Gray L. J., Khunti K., Mohan V., Hughes R. (2013). Real-world factors affecting adherence to insulin therapy in patients with type 1 or type 2 diabetes mellitus: a systematic review. *Diabetic Medicine*.

[24] Sarbacker G. B., Urteaga E. M. (2016). Adherence to insulin therapy. *Diabetes Spectrum*.

